# Nutrition and the Immune System: A Complicated Tango

**DOI:** 10.3390/nu12030818

**Published:** 2020-03-19

**Authors:** Carina Venter, Stefanie Eyerich, Tara Sarin, Kevin C. Klatt

**Affiliations:** 1 Section of Allergy & Immunology, School of Medicine, University of Colorado Denver, Children’s Hospital Colorado, Anschutz Medical Campus, 13123 East 16th Avenue, Aurora, CO 80045, USA; Tara.Sarin@childrenscolorado.org; 2 Center of Allergy and Environment (ZAUM), Technical University and Helmholtz Center Munich, Biedersteinerstrass 29, 80802 Munich, Germany; stefanie.eyerich@tum.de; 3 USDA/ARS Children’s Nutrition Research Center, Baylor College of Medicine, Houston, TX 77030, USA; kcklattphd@gmail.com

**Keywords:** nutrition, omega-3 fatty acids, fiber, immune system, microbiome

## Abstract

Enthusiasm exists for the potential of diet to impact the immune system, prevent disease and its therapeutic potential. Herein, we describe the challenge to nutrition scientists in defining this relationship through case studies of diets and nutrients in the context of allergic and autoimmune diseases. Moderate-quality evidence exists from both human intervention and observational studies to suggest that diet and individual nutrients can influence systemic markers of immune function and inflammation; numerous challenges exist for demonstrating the impact of defined diets and nutrient interventions on clearly influencing immune-mediated-clinical disease endpoints. A growing body of evidence suggests that further consideration of dietary patterns, immune system and gut microbiome composition and function, and subsequent epigenetic modifications are needed to improve our understanding of diet–immune system interactions.

## 1. Introduction

The complexity of the interaction between nutrition and immunology is vast. An individual’s overall nutrition status, state of nourishment, and pattern of food intake (comprised of foods, nutrients and non-nutritive bioactive compounds) impact the functioning of the immune system; this impact can occur at the level of physical barriers (e.g., skin, intestinal mucous membranes), the microbiome, the innate immune system (e.g., macrophage function and polarization) and the adaptive immune system (e.g., T- and B-cell function). Conversely, the immune system impacts nutrition metabolism and needs, and influences the physiological response to food. This complex relationship between nutrition, diet and the immune system underlies the rationale behind this current review. Within, we will describe the developing field of nutritional immunology through case studies of the relationship between nutrition and the immune system.

## 2. Approaches to Studying Nutrition and the Immune System

Assessing the bidirectional relationship between diet and the immune system can be undertaken utilizing multiple approaches. In human intervention studies, investigators have assessed the impact of bioenergetic status [1], isolated nutrients [2,3,4,5,6], and dietary patterns, such as the Mediterranean Diet [7,8], in both controlled feeding and free-living intervention studies on numerous indices of immune function (e.g., circulating cytokines, high-sensitivity C-reactive Protein, antibodies, tissue-specific transcriptomes). To complement such intervention approaches, a growing body of literature, utilizing observational study designs, has assessed dietary intakes via self-reported measures and circulating biomarkers, and assessed their associations with similar immune function outputs, as well as disease endpoints (e.g., allergy incidence, chronic disease risk). Such investigations have occurred in a variety of populations, including pregnant women and young infants, adults, individuals with chronic disease, metabolic syndrome, allergic, inflammatory and/or autoimmune diseases. Typically, these clinical observations follow and are complemented by investigations in laboratory animals and cultured cells to provide mechanistic insights, although important differences in immune system development and function [9,10] and lack of in vivo interactions limit the direct translation of findings in animal studies to humans.

It is critical to note that, at present, few large, randomized controlled trials exist with clinical endpoints (e.g., event reduction; disease remission) that demonstrate an impact of diet on immune-mediated disease risk. In some instances, such as the case of early dairy protein exposure and risk of beta-cell autoimmunity, bioplausible hypotheses have not been confirmed in large clinical trials [11]. The difficulties facing nutritional immunology and the caveats of relying on surrogate endpoints are made further evident by the long history of testing the inflammation-atherosclerosis hypothesis; researchers have spent decades employing numerous anti-inflammatory agents prior to demonstrating an effect of interleukin (IL)-1 beta inhibition on cardiovascular event risk reduction. While enthusiasm for nutrition and immune-mediated disease risk abounds, careful consideration of the nature and quality of the data are paramount. 

## 3. Nutrition and Immune-Mediated Disease Risk

Dietary intake throughout the life span ranging from conception to old age, has been hypothesized to play a significant role in the development, management and treatment of noncommunicable diseases including allergic diseases, cancer, diabetes, and cardiovascular disease. Notably, such noncommunicable diseases have well-described immunopathological processes, raising the possibility that immunomodulatory aspects of diet may causally influence disease risk and management. The incidence of immune-mediated diseases is elevated in Westernized countries where the burden of such diseases is high, typically ascribed to common dietary components such as the high intakes of total calories, fat and added sugars, low intakes of fiber, and imbalanced fatty acid composition of the diet. Consistent with these ecological correlations, specific nutrients and dietary patterns have been associated with lower risk of allergic and chronic inflammatory disease development [12,13]. A growing body of preclinical and clinical literature has emerged describing the impact of individual dietary components and dietary patterns on markers of immune function, potentially underlying some of these associations (Table 1). A thorough description of the field of nutritional immunology is beyond the scope of a single review; thus, to highlight specific ways that researchers have studied this relationship, below we describe both allergic and autoimmune diseases and the possible impact of nutrition on disease incidence and management.

## 4. Allergic Disease

Allergy is an immune-mediated reaction, specific to an encounter with a range of allergens such as foods or environmental exposures [56]. It can present in almost every organ and launch a range of symptoms such as anaphylaxis, urticaria, angioedema, allergic rhinoconjunctivitis, allergic asthma, allergic vasculitis and atopic dermatitis (eczema) [57]. The four most common allergic diseases are referred to as eczema, food allergy, asthma and rhinitis [58]. Eczema and food allergies usually develop in infancy, and affected infants often progress onward, developing asthma and allergic rhinitis in a sequential manner called the ‘allergic march’ [59]. The immunological processes underpinning allergic diseases presents in two phases—the sensitization phase and the effector phase. During the sensitization phase, naïve T cells recognize an allergen and differentiate into T helper (Th) 2 effector cells that secrete IL-4, IL-5 and IL-13 that, in turn, induce allergen specific immunoglobulin (Ig) E production by B-cells [60]. Allergen-specific IgE binds to the high-affinity receptor (FcεRI) on mast cells and basophils completing the sensitization phase. When the immune system encounters the allergen again the effector phase is induced. Here, the allergen binds to surface-bound IgE and cross-links two FcεRI receptors on mast cells or basophils leading to consecutive release of pre-formed mediators such as histamine and prostaglandin that provoke the typical allergic symptoms described above. Whereas T-, B-, mast cells, eosinophils and basophils represent essential cellular mediators during sensitization and effector inflammation, a defective epithelial barrier has been more recently shown to allow penetration of allergens, bacterial toxins and other particles, leading to inflammation and release of IL-25, IL-31, IL-33 and thymic stromal lymphopoietin (TSLP) [61] that stimulate the production of allergen-specific IgE, the recruitment of eosinophils and other inflammatory cells, mucous production and reduce contraction of smooth muscles [59]. Thereby, allergic reactions are induced by a complex interaction of cells and mediators of both, innate and adaptive immunity.

### Overall Diet 

The best evidence that overall nutritional intake may play a role in allergy prevention comes from the studies focusing on diet diversity in infancy. The European Academy of Allergy and Clinical Immunology (EAACI) position paper [62] regarding diet diversity and prevention of allergies concludes that diet diversity in infancy may be associated with reduced allergy outcomes in childhood and may be beneficial given low to no risk of harm [62]. Diet diversity is defined as the number of different foods or food groups over a reference period, and should ideally include frequency of consumption and the health value of the food. It is thought that diet diversity may influence allergy outcomes via its effect on the microbiome and immune system. This alteration in the immune system may be mediated through a multitude of immune antigen tolerance mechanisms including T and B regulatory cells, immune regulatory cytokines and suppressed IgE antibodies as demonstrated in other allergen tolerance models [62]. So far, all studies into diet diversity have been conducted in infancy [21,62]. A recent study indicates that both increased diet diversity and allergen diversity in the first year of life, is associated with a reduced risk of developing food allergy over the first ten years of life [21]. No studies focusing on diet diversity during the other life stages such as pregnancy and later life and allergy outcomes have been published.

Data on other diet patterns, in particular, the Mediterranean diet, give some evidence that eating according to these diet patterns in pregnancy may reduce wheeze or eczema in the infant. No study focusing on diet patterns in infancy, and other life stages on allergy outcomes have been conducted [62]. 

## 5. Single Nutrients

### 5.1. Omega-3 and Omega-6

**Figure 1:**
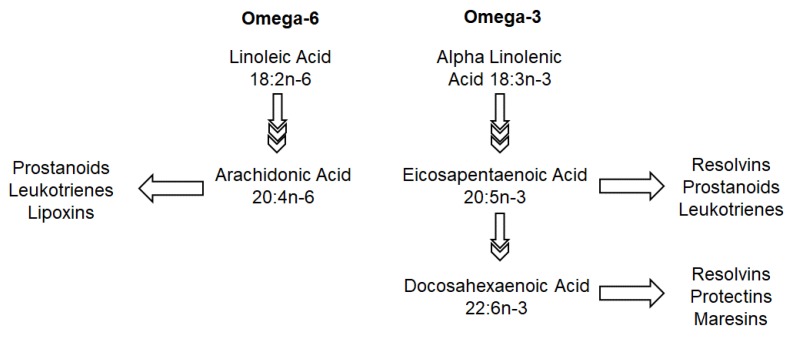
Omega-6 and Omega-3 fatty acid metabolism.

Responses to immune stimuli require both the initiation and resolution of an immune response [63]. Central to this coordinated response are polyunsaturated fatty acids (PUFAs) of the omega-6 and omega-3 series that serve as substrates for the synthesis of signaling molecules, including eicosanoids and docosanoids (Figure 1) [64]. Diet serves as a source of a variety of key PUFAs, including the essential fatty acids, linoleic acid (18, 2*n*-6) and alpha-linolenic acid (18, 3*n*-3), as well as their longer chain and more highly unsaturated products arachidonic acid (arachidonic acids (AA); 20, 4*n*-6), eicosapentaenoic acid (eicosapentanoic acid (EPA; 20, 5*n*-3)) and docosahexaenoic acid (docosapentanoic acid (DHA) 22, 6*n*-3). Nutritional manipulation of the membrane content of PUFAs, particularly of the longer chain omega-3 series (LCn3PUFA), has generated great interest due to their enrichment in various immune cell types, as well as their ability to both reduce AA contents of the membrane and antagonize AA metabolism. Several eicosanoid derivates of AA, including prostaglandin E2 and 4-series leukotrienes, have been implicated in promoting sensitization to allergens and increased disease severity, and thus, adequate LCn3PUFA status during both early immunological development and at the time of established immune–antigen interfacing may modify disease risk. 

The EAACI position paper suggests that, in patients with the lowest preexisting levels of long-chain polyunsaturated fatty acids (LC-PUFAs), supplementation may be beneficial in allergy prevention, particularly in those with low levels of omega-3 fatty acids [65]. Such protective effects are most commonly seen in pregnant and lactating women, whereby increasing maternal and breast milk LCn3PUFA levels are associated with reduced risk of AD and development of food allergies, although significant heterogeneity in the evidence base exists [65]. Sources of this heterogeneity need further consideration, but likely relate to the dose of LCn3PUFA used in clinical trials, baseline and achieved LCn3PUFA status, the timing of supplementation, common genetic variants throughout fatty acid metabolism and immunity, and microbiome composition [65]. Current recommendations emphasize an individualized approach to nutrition and further, well-designed human studies with challenge-proven food allergy are necessary [66].

LCn3PUFA have received substantial interest for not only their role in prevention of immune-mediated disease, but also their impact on reducing the severity of established disease. In addition to the role of EPA and DHA in antagonizing AA metabolism, they serve as substrates for the production of less potent eicosanoids (from EPA) and specialized pro-resolving mediators (SPMs) [64,67]. SPMs include resolvins (D- and E-series produced from DHA and EPA, respectively), protectins, and maresins (derived from DHA), and appear to act primarily by inhibiting the recruitment and activation of multiple immune cell types, conferring pro-resolving and analgesic properties [68,69]. These novel compounds provide further enthusiasm for omega-3 fatty acids in managing immune-mediated diseases and underlie the enthusiasm for EPA/DHA supplementation in autoimmunity.

Of the available literature base in autoimmune diseases, a substantial body of randomized controlled trials testing the impact of LCn3PUFA, primarily EPA and DHA mixtures, exist for both inflammatory bowel diseases and rheumatoid arthritis. To date, the literature in inflammatory bowel disease has been disappointing. Available systematic reviews and meta-analyses of controlled trials [70,71,72,73,74] consistently show that LCn3PUFA supplementation does not prolong states of disease remission in Ulcerative Colitis or Crohn’s Disease, and there is high uncertainty due to low quality about the effect of LCn3PUFA in active disease. Notably, supplementation is not without side effects, with patients exhibiting an increased risk of diarrhea and upper gastrointestinal side effects. The complexity of the immunopathology of Ulcerative Colitis and Crohn’s Disease, involving impaired mucosal barrier function, varied cell types of the innate and adaptive immune system and their secreted mediators, gut microbial composition and the response to other various luminal factors make it difficult to explain why LCn3PUFAs have likely failed to influence clinical disease. Recent studies in animal models employing the dextran sulphate sodium model of colitis additionally suggest that high-dose LCn3PUFA worsen disease phenotypes when started just prior to dextran-sulphate-sodium provision [75], conflicting with existing evidence from transgenic Fat1 mice [76], capable of synthesizing their own omega-3 fatty acids, that have demonstrated significant protection from colitis. Such data suggest that the degree of tissue omega-3 status saturation, the relative impact on other fatty acid species, and timing of increased omega-3 status require further investigation to determine any potential efficacy of omega-3 fatty acids in humans with inflammatory bowel disease.

The impact of LCn3PUFA in rheumatoid arthritis is more promising. Supplementation has been shown to reduce leukotriene B4 [77], a chemotactic factor released from neutrophils that is a key driver of inflammatory arthritis [78]. Consistent with this reduction in causal pathophysiological mediators of disease, systematic reviews and meta-analyses of small clinical trials in rheumatoid arthritis consistently identify reduced non-steroidal anti-inflammatory use, improved pain, joint tenderness and improved physical functioning [79,80]. Effective doses of LCn3PUFA in shorter term supplementation trials have tended to be in the pharmacological range (>2.5 g/d EPA + DHA), though self-reported intakes of food sources of omega-3 fatty acids are associated with improved self-reported disease scores [78,81]. Large, confirmatory trials in patients with rheumatoid arthritis are needed for LCn3PUFA status monitoring and supplementation to become standard of care; indeed, the available evidence leaves many questions about the optimal dose, duration, and composition of omega-3 fatty acids, their effectiveness alongside modern medications (e.g., TNF-alpha inhibitors), and their role in sero-positive vs sero-negative disease states.

### 5.2. Fiber

In line with the role of nutrition on the immune system, we have more a glimpse than a profound understanding of how the microbiome can be beneficially influenced by dietary compounds. However, it is well appreciated that fibers as non-digestible parts of fruits, vegetables and cereals are an important energy source for bacteria that, by fermentation, lead to the production of short-chain fatty acids (SCFA) as essential nutrients for humans. In numerous studies using different fiber interventions, fibers have been attributed to maintain intestinal homeostasis by enhancing epithelial barrier function, inhibiting pathogen-induced cytotoxicity and preventing colonization with pathogenic bacteria. 

Despite most studies having been performed in in vivo animal models, there is early proof that fiber intake can also ameliorate pathology in humans in various organs. A high-fiber diet favors microbial diversity and production of SCFA and prevents the fermentation of less favorable substrates such as proteins and amino acids, leading to a reduced risk for colorectal cancer and Crohn’s disease [82,83]. In addition, SCFA are absorbed and distributed systemically via blood circulation and thereby, may also prevent pathologies outside the gut. Patients suffering from asthma or cystic fibrosis present with a reduced microbial diversity in the gut leading to a shift from SCFA production to lipid, amino acid and carbohydrate metabolism [84,85]. A long-term fiber-rich diet has been shown to improve lung function and to lower the risk for COPD [86,87]. In addition to this microbial gut–lung axis, evidence exists that the gut–brain axis can also be influenced by fibers beneficially. Studies using dietary supplementation with Glucose-oligosaccharides or human milk oligosaccharides indicated a reduction of anxiety scores in irritative bowel syndrome patients and acetate influenced appetite by enhancing the production of regulatory neuropeptides [88,89]. Furthermore, people following a Mediterranean diet (30 g fiber/day) have a lower risk for type-2 diabetes and patients at risk for cardiovascular disease show lower incidence of events, highlighting the beneficial effects of fibers on metabolic syndrome [90,91,92]. Mechanistically, high-fiber diets may influence immune-mediated diseases, e.g., by the impact of SCFAs on signaling through G-protein coupled receptors (GPR), namely GRP41, GPR43 and GPR109A [93,94,95], that are highly expressed on a variety of tissues including myeloid-derived immune cells. Additionally, acetate and butyrate, two common SCFAs, exhibit the capacity to inhibit histone deacetylase activity [95,96], broadly influencing chromatin structure and the epigenetic state of the cell. Further in vivo animal work and human studies are needed to assess the contribution of epigenetic modifications to immune cell function, though a significant body of work suggests that HDAC inhibition in epithelial cells is critical for barrier function and influencing the immune response [97]. 

This highlights the potential of fibers as an important tool for disease prevention [98]. The challenge in the future will be to integrate fibers into our diets and efforts should be undertaken to educate children (and adults) to at least reach the recommended intake of 25–31 g fiber/day or even higher amounts (Table 2). However, personalized approaches also need to be implemented as one-size does not fit all and, under certain underlying diseases (e.g., inflammatory bowel disease) and prompt increase of dietary fiber content, unwanted side effects of a high-fiber diet such as flatulence, stomachaches, constipation and diarrhea might occur.

## 6. Future Directions

The relationship of diet to allergy prevention and autoimmune disease management illustrates the complexity of the questions at hand, in addition to the limitations of the available evidence base. Numerous diet-x-immune-x-microbiome interactions are likely relevant, in addition to the heterogeneity in patient and at-risk population characteristics. Such complexity obscures clear identification of signals for dietary modification, although many bioplausible relationships exist. In addition to well-controlled feeding trials and rigorous prospective cohort studies, below we discuss additional considerations for advancing this field of study in allergy and autoimmune diseases, with relevance to additional immune-mediated pathologies.

### Accounting for Dietary Patterns

Individual nutrient approaches allow for clear tests of hypotheses about their relationship to disease risk and management. However, individual nutrients are consumed in the context of overall dietary patterns and interpretation of results must be appropriately contextualized in this manner. The effect size of individual nutrients is often small and thus, diets differing in multiple nutrients may be of interest to assess their impact on disease outcomes; however, some nutrients target common pathways and it is difficult to know whether multi-nutrient interventions will have additive, interactive, or antagonistic effects on each other. Similar to the trials of the Dietary Approaches to Stop Hypertension (DASH) diet, and DASH-Sodium trials [100,101], trials of whole dietary patterns thought to influence the immune system and disease risk, concomitantly modifying individual nutrients hypothesized to strongly influence outcomes of interest, are warranted in the field of nutritional immunology. Further characterization of the total diet and derivation of dietary patterns that appear protective against the development of immune-related diseases may further advance the field and provide the basis for future, rigorous trials and preclinical investigations to identify novel, immunomodulatory dietary components. 

## 7. The Way Forward

**Figure 2:**
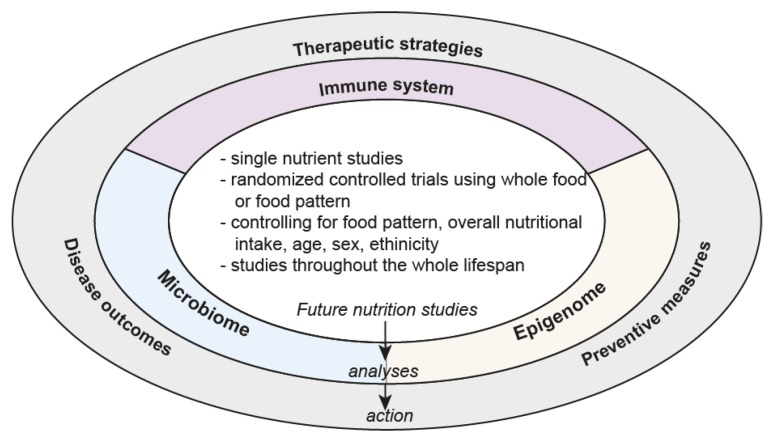
Future direction of nutritional research.

Nutritional interventions contain the potential to prevent or improve disease. However, until reliable recommendations can be given to physicians and patients, mechanistic studies of nutritional patterns and/or single nutrients on immune function, microbiological and epigenetic changes have to be performed to fully understand the role of nutrition on disease outcomes (Figure 2, Box 1). In addition, studies are needed that comprehensively link all three components together and determine causal mediators of nutrient-induced physiological changes and health outcomes.

To study these multifactorial exposures requires coordinated interdisciplinary efforts, but performed well, can lead to a global change in the prevention and management of non-communicable diseases. Addressing the current state of the literature and undertaking such interdisciplinary efforts will require a commitment from the broader scientific community to funding well-powered, controlled feeding studies in both preclinical designs and human trials across diverse populations.

Box 1 **The way forward in nutrition research.**

1. Randomized controlled trials using food patterns/whole diets controlling for environmental factors such as air quality, water content, exposure to sunlight.
2. Single-nutrient studies controlling for other dietary/environmental factors
3. Mechanistic studies focusing on how food impacts on the immune system, microbiome, epigenome and genome and interaction of these components
4. Trials controlling for sex, ethnicity and race
5. Improved tools to measure dietary intake

## 8. Conclusions

Clinicians strive to provide answers to their patients, but there are many unanswered questions related to the role of nutrition on disease prevention [102,103]. Two of the nutrients often studied (but not exclusively studied) in their relation to the immune system are LCn3PUFA and fiber, but much information is required before disease-specific recommendations can be made with high confidence. Perhaps the answer does not lie in single nutrients alone; more focus on randomized controlled trials modifying multiple immune-modulating dietary components as part of a broader dietary patterns should be conducted. Until clear answers are found, the nutrition world will continue to, often contentiously, argue for specific dietary recommendations on low-quality evidence. Once we truly know how the enjoyment of eating can be linked with evidence-based medicinal outcomes, we can then focus on effective implementation of such interventions. 

## Figures and Tables

**Table 1:** Impact of diet patterns/types (A), nutrients (B) other nutritional factors (C) and food preparation/production (D) on gut barrier function, inflammation and the microbiome.

**(A) Diet Patterns/Types**		
**Gut Barrier Function**	**Inflammation**	**Microbiome**
		**Overall diet** can affect production of inflammatory/anti-inflammatory metabolites by microbiome [14] A “tolerant” gut microbiome may reduce expression of IL-33 and TSLP and may protect against sensitization to food allergens [15]. (mainly murine models)
**Western type diet:** high saturated/trans fat and protein; low fiber: may affect goblet cell function and reduce mucus layer [16] in murine models		**Western-type diet:** high saturated/trans fat and protein; low fiber: Can lead to low microbial diversity. Reduces populations in the phylum *Bacteroidetes* and increases *Firmicutes* and *Proteobacteria in murine models* Reduces populations in the phylum *Bacteroidetes* and increases *Firmicutes* and *Proteobacteria in human studies* [17,18,19,20]
		Higher **diet diversity** is associated with a more diverse gut microbiome in human studies. Diet diversity has been reported to prevent allergic disease [21], but it’s direct role in the management of food allergy has not been investigated [22,23,24] High levels of butyrate and proprioate and a diet high in fermented foods, fruit and vegetables and fish infancy is also associated with reduced allergy outcomes [25].
**(B) Nutrients**		
**Gut Barrier Function**	**Inflammation**	**Microbiome**
	**Vitamins**–particularly Vitamin A and B9, affect T-regulatory cell function and act as ligands [26,27] as demonstrated in murine models.	
**Omega-6 fatty acids:** enhance tight junctions [28] in murine models	**Long chain poly-unsaturated fatty acids** particularly omega-3 fatty acids: suppress allergic inflammation via its effect on resolvin D1 and peroxisome proliferator-activated receptors (PPAR) in murine models [29] Can also affect the FADS1 genotype (rs174550) [18,19]	
Acid; Docosahexaenoic Acid (long chain omega 3 fatty acids) [30,31]	hsCRP, IL-6, TNF-alpha [30,31]	
Dietary Sodium [32,33,34,35]	Increased Th-17/T-regulatory ratio	
Dietary Genistein and Daidzein (soy isoflavones) [36]	Decreased CRP	
	**Amino acids:** play and important role in cell wall structures in murine models [37]	**Amino acids:** Certain amino acids such as D-tryptophan may affect the production of bacterial products that can positively affect immune mediated diseases as shown in murine [37].
**(C) Other Nutritional Factors**		
**Gut Barrier Function**	**Inflammation**	**Microbiome**
**Advanced glycosylated end products (AGEs):** may affect epithelial cell function as shown in murine models [38,39]	**AGEs** may affect inflammatory processes, particularly via its effect on IL-33 and TSLP as shown in mice [40]	**AGEs:** may negatively affect the microbiome composition as shown in a rodent model [41].
**AGE content of foods may be affected by sugar content,** grilling or roasting meats, high fat content, highly processed foods, fruit juices [42], high fructose corn syrup [43,44] and fizzy drinks [45]. Steaming, boiling, slow-cooking and using acids when cooking can reduce the amounts of AGEs produced [40].		
		**Prebiotics:** selectively stimulate the growth of beneficial bacteria and might offer protection against effects of AGEs as shown in a human trial [46]
**Fiber:** Short chain fatty acids (SCFAs) are produced through the fermentation of polysaccharides and improve gut barrier function via its effect on IL-22 (promoting mucus production) as shown in murine models [47,48]	**Fiber:** Short chain fatty acids (SCFAs) are produced through the fermentation of polysaccharides and reduce allergic inflammation as shown in murine models [47,48]	
		Polyphenols: Increase gut microbial diversity [49,50] indicated by human studies
**Emulsifiers** e.g., polysorbate 80 and carboxymethylcellulose may destroy the epithelial mucous layer in the gut as shown in mice [51]	**Emulsifiers** e.g., polysorbate 80 and carboxymethylcellulose, promote inflammation as shown in mice [51]	**Emulsifiers** e.g., polysorbate 80 and carboxymethylcellulose, alter gut microbial composition as shown in mice [51]
**(D) Food Preparation/Production**		
**Gut Barrier Function**	**Inflammatory Processes**	**Microbiome**
		**Uncooked foods,** cooking methods and processing can affect the natural microbial load of foods—Fresh foods contain their own microbiome, including nonpathogenic bacteria (e.g., Lactobacillus plantarum on fruits and vegetables) [52,53]
		Phthalates (a chemical compound from packaging) found in fast foods [54] and some initial data from murine models indicate that it may reduce microbioal diversity in the gut [55]

AGEs: advanced glycytion end products; CRP: C-reactive Protein; IL: interleukin; FADS1, fatty acid desaturase 1; TNF: tumor necrosis factor; Th: T helper cell; TSLP: thymic stromal lymphopoietin; SCFAs: short-chain fatty acids. Bold indicates the particular nutrient factor studied.

**Table 2:** Institute of Medicine guidance on fat and fiber intake [99].

Calorie Level(s) Assessed		1000	1200	1400, 1600	1600	1800	1800	2200, 2800, 3200
**Macronutrients**								
Total fat, % kcal	AMDR	30–40	25–35	25–35	25–35	25–35	25–35	25–35
Saturated fat, % kcal	DGA	<10%	<10%	<10%	<10%	<10%	<10%	<10%
Linoleic acid, g	AI	7	10	10	10	12	11	16
Linolenic acid, g	AI	0.7	0.9	0.9	1	1.2	1.1	1.6
Fiber	14 g/1000 kcal	14	16.8	19.6	22.4	25.2	25.2	

AI = Adequate Intake, Intake Level, AMDR = Acceptable Macronutrient Distribution Range, DGA = *2015*–*2020 Dietary Guidelines* recommended limit; 14 g fiber per 1000 kcal = basis for AI for fiber.
